# Research Involving Health Providers and Managers: Ethical Issues Faced by Researchers Conducting Diverse Health Policy and Systems Research in Kenya

**DOI:** 10.1111/dewb.12130

**Published:** 2016-10-04

**Authors:** Sassy Molyneux, Benjamin Tsofa, Edwine Barasa, Mary Muyoka Nyikuri, Evelyn Wanjiku Waweru, Catherine Goodman, Lucy Gilson

**Keywords:** health policy and systems research, ethical issues and dilemmas, qualitative research, governance research, relationships, power

## Abstract

There is a growing interest in the ethics of Health Policy and Systems Research (HPSR), and especially in areas that have particular ethical salience across HPSR. Hyder et al (2014) provide an initial framework to consider this, and call for more conceptual and empirical work. In this paper, we respond by examining the ethical issues that arose for researchers over the course of conducting three HPSR studies in Kenya in which health managers and providers were key participants. All three studies involved qualitative work including observations and individual and group interviews. Many of the ethical dilemmas researchers faced only emerged over the course of the fieldwork, or on completion, and were related to interactions and relationships between individuals operating at different levels or positions in health/research systems. The dilemmas reveal significant ethical challenges for these forms of HPSR, and show that potential ‘solutions’ to dilemmas often lead to new issues and complications. Our experiences support the value of research ethics frameworks, and suggest that these can be enriched by incorporating careful consideration of context embedded social relations into research planning and conduct. Many of these essential relational elements of ethical practice, and of producing quality data, are given stronger emphasis in social science research ethics than in epidemiological, clinical or biomedical research ethics, and are particularly relevant where health systems are understood as social and political constructs. We conclude with practical and research implications.

## Background

There is increasing recognition in low‐and‐middle income countries (LMICs) of the importance of health systems in achieving health‐related development *goals,* and of the constraints related to health system short‐falls and weaknesses.^1^ The recent Ebola virus disease outbreak in western Africa has been a devastating reminder of how an epidemic can proliferate rapidly and pose huge problems in the absence of a strong health system capable of a rapid and integrated response.^2^ As Kieny et al point out, ‘a strong health system decreases a country's vulnerability to health risks and ensures a high level of preparedness to mitigate the impact of any crises’ (2014).

Research has an important role to play in strengthening health system performance and public health.^3^ There are multiple definitions of health systems research, many of which overlap with definitions of operational research and implementation research. In this paper we use the extended term Health Policy and Systems Research (HPSR) in recognition of the interconnections between health policy and systems, and the social and political nature of the field.^4^ Although HPSR is a broad field, there is emerging consensus on key areas of focus including: the performance of health systems and their subcomponents (resources, organizations, and services); how links among the subcomponents shape performance, and what forces influence those links; and how to strengthen health system performance over time.^5^ Importantly, HPSR is recognized as a hybrid, or ‘trans‐disciplinary’ field, drawing on different disciplinary traditions and methodological approaches. It is also recognised as applied research that is undertaken with an orientation towards influencing policy and wider action to improve the performance of health systems.^6^

In parallel with an emerging consensus of some key features of HPSR, there is a growing interest in and debate over the ethics of HPSR.^7^ These developments are linked in part to the increasing support by funders for HPSR in response to recognition of its importance, including from the World Health Organisation, the Gates foundation, the Department for International Development (DFID), the Economic and Social Research Council (ESRC), the Medical Research Council (MRC) and Wellcome Trust. In addition to the many general health research ethics guidelines and recommendations that are relevant for HPSR, there are guidelines for particular types of research that are tailored to specific forms of HPSR, such as for participatory research,^8^ implementation research,^9^ cluster trials^10^ and learning health systems.^11^ Across these documents and associated commentaries, it is suggested that certain kinds of ethical issues may be particularly relevant in HPSR, and that ethics review committees are not as well equipped to identify and evaluate these issues as they are for more familiar forms of biomedical research such as clinical trials. Hyder et al^12^ have published an initial framework to facilitate discussion on ethical issues and appropriate oversight of health systems research, with a focus on LMICs. However as they and others note, challenges in building up an understanding of the ethics of health systems research as a specific theme include the diverse range of studies and disciplines involved, what is often a grey zone between research and non‐research, and the many overlaps of issues with other types of health research.^13^ They argue for greater conceptual work and empirical research aimed at better understanding health systems research ethics.^14^

There are relatively few published papers that explore the ethical issues faced over the course of conducting HPSR, especially from LMICs. This is an important gap: an understanding of the everyday practice of HPSR in diverse social, economic and political contexts can help us to move closer towards a more ‘situated ethics’ of research, in which the relevance and application of ethical principles and guidelines for different studies and contexts, is considered.^15^ In a previous paper we reviewed the ethical issues faced during the conduct of household based studies conducted in two very different social contexts.^16^ We organised our findings based on Emanuel et al's framework^17^ which draws on a diverse range of ethics regulations and guidelines to consider eight principles in planning and reviewing clinical research in developing countries: collaborative partnership, social value, scientific validity, fair selection of study population, favourable risk‐benefit ratio, independent review, informed consent and respect for recruited participants and study communities.

In our previous paper, we showed the potential value of the Emanuel framework and of incorporating ideas from social science research guidelines into ethics review and practice in household‐based multi‐method studies. Although there is a shorter tradition of established research ethics in the social sciences,^18^ guidelines are increasingly being developed. There is significant diversity and debate across the social sciences regarding ethical guidance. However, broad differences between biomedical and social science guidelines include^19^: 1) Less codification of ethics in the social sciences compared to biomedicine. Thus, for example, in biomedicine there are usually more elaborate requirements in place with regards to ensuring informed consent, confidentiality, individual benefits for participants and avoidance of harm; 2) greater attention in the social sciences to reflexivity, interpersonal relationships, and the role of trust and power imbalances inherent in many research relationships; and 3) a more central focus in the social sciences on the political implications of the research endeavour.

In this paper we build on this earlier work through focusing on ethical issues that emerged over the course of three HPSR studies in which health managers and providers at the district, county or facility level were key study participants or communities. All three studies included observations and individual and group interviews, and focused on a crucial but neglected topic in HPSR: the micro‐level processes of governance at sub‐national and local levels, and the implications for health system resilience and responsiveness to local communities.^20^ All three studies had a broad goal of contributing to strengthening equity in health and health systems. In sharing our experiences we focus on the ethical issues and dilemmas we faced as researchers as opposed to examining ethical practice among health workers or managers, although – as will be shown ‐ perceptions of the latter inevitably influence researchers’ experiences. Also of note is that we do not believe that many of the issues we faced are in practice specific to HPSR, but are likely to be of relevance for a diverse range of studies in LMICs.

## Methods

We are all researchers involved in the conduct of governance studies funded by DFID as part of a multi‐country health systems research consortium on Resilient and Responsive Health Systems (RESYST; resyst.lshtm.ac.uk). The three studies of interest were or are being conducted in Kenya. They have varying levels of action research/interventions incorporated into them, but all aim to understand the performance of elements of health systems, complex influences on this performance, and how to strengthen performance over time.

An overview of the studies is presented in Table 1, but briefly:

**Table 1:** Summary of the three studies of interest

Study	HSSF – study 1	Hospital priority setting – study 2	‘Learning sites’ ‐ study 3
Research objectives	Track the implementation and perceived impact of an innovative direct facility funding mechanism for peripheral health facilities	Understand priority setting processes in hospitals	District level action learning and reflective practice in South Africa and Kenya
Nature of health system intervention being studied	A national financing mechanism with associated accountability mechanisms	No specific new ‘intervention’; documenting routine activities	No formal intervention specified at the outset, but an intention for managers and researcher to change and track micro‐governance processes as appropriate
Research methods	Mixed methods, including a survey and a range of qualitative methods (primarily individual and group interviews)	Qualitative: ethnographic observation, in‐depth formal and informal interviews	Qualitative with action elements: (participant) observation, in‐depth formal and informal interviews and reflective practice with health managers and providers, and community members
Research setting(s)	5 districts across Kenya	Coastal Kenya – 2 hospitals	Kilifi County in Kenya
Funders	DFID, Wellcome Trust, DANIDA	DFID, Wellcome Trust	DFID, Wellcome Trust
Dates	2012‐2014	2013‐2015	2013‐ongoing

**Table 2:** Summary of the outcomes

General areas	Challenges faced and where examples illustrated
Informed consent and respect for recruited participants and communities.	Information and consent processes Perception of our work being some kind of audit or check (Box 2) How much choice do some health managers and providers really have about involvement in our research? Is information we gain through informal interactions or our other capacities (or hats) covered by our consent processes? (Box 3) How long a period can our consent processes cover? Apparent perception that we can and should assist to deal with day to day problems observed or otherwise identified. *Respect to participants and communities beyond consent* How to respond when being informed about or observing apparently ‘unethical’ behaviour (Boxes 1 and 2) Individual informed consent in terms of fixed messages given formally to all potential participants at the outset of the study is only one part of a much wider set of interactions which show respect to diverse participants. Regular interaction, discussion and reflection with key actors wherever possible is essential. Ensuring that all actors in the research team understand and ‘buy into’ the research and research approach is essential to asking the right questions, building good quality relevant data, and minimising unrealistically high expectations. Feedback to collaborators and actors at different levels of the system is essential and may often require informal interaction, and being able to respond to requests for information and engagement with very short notice.
Social value and risk‐benefit ratios.	Individual risks and benefits are not as obviously and automatically incorporated into HPSR in the way they often are for epidemiological or clinical studies. Careful consideration and planning is needed to ensure that relationships are not harmed, and power imbalances not exacerbated. A dilemma is how to counter‐balance this cautionary approach against a transformative agenda to raise awareness about and reduce damaging imbalances in an effort to strengthen equity. Where the intention is to improve equity between actors, there needs to be careful planning and tracking of activities over time to ensure that there are no unintended perverse outcomes to the contrary. Specifically, there may be particular concerns regarding breach of confidentiality, where individuals, facilities or regions may be easily identifiable, with negative implications for future (transformative) research.
Independent review	Given the importance of committees as gatekeepers of research, and the challenges faced in terms of the resources available and the range and number of studies they have to review, we support others in advocating for strengthened support to these committees in their review of HPSR, including assessing qualitative research.

‐ The **health sector services fund (HSSF) study** was a mixed methodology study, including quantitative elements, aimed at tracking the implementation and impact of an innovative direct facility funding mechanisms in public primary health care facilities (health centres and dispensaries).^21^ Data were collected in five districts across the country primarily from facility and district health managers, and community representatives with a formal role of assisting with the management of those facilities (health facility committee members). The researchers did not have any role in implementing what was a national government finance initiative; they were simply tracking the ground realities of its implementation in a once off interaction with the majority of research participants. Thus this study is a relatively straightforward descriptive study.
‐ The **hospital priority setting study** was a nested case study design, where two public hospitals in coastal Kenya were selected as cases and three priority setting processes examined as nested cases. Data were collected over a seven month fieldwork period using in‐depth interviews, document reviews, and non‐participant observations, with multiple interactions with key research participants. The research participants were primarily senior and mid‐level hospital managers and administrators (some also with clinical responsibilities), and there was no specific intervention introduced by the researchers; researchers were documenting routine activities. This study is a more in‐depth anthropological study conducted over time in only two facilities.
‐ The **learning sites study** is on‐going and is an action approach to learning in which the intention is for health system managers and HPSR researchers to work together to identify research questions and to document initiatives aimed at positively strengthening micro‐governance processes. Potential interventions to examine range from small but critical changes in everyday governance practice (such as how meetings are organised) through to more formal interventions such as a change in finance policy for the county. Researchers and health managers at various levels interact regularly both formally and informally, with the specific intention of breaking down the researcher‐participant distinction. This study is a relatively innovative and embedded, long‐term approach to governance research.^22^

All three studies were reviewed for scientific validity and ethics and approved in advance at institutional and national level in Kenya, and by the London School of Hygiene and Tropical Medicine in the funding country (UK).

As researchers involved in the studies several of the authors^23^ organised two specific meetings, each of two hours, to formally reflect upon the ethical issues and challenges they have faced in their work, if and how they have attempted to resolve these, with what apparent implications. We loosely defined an ethical problem as ‘a problem or situation that requires a person or organization to choose between alternatives requiring ethical analysis’, as opposed to a problem where it is clear what should be done, but a practical solution is needed. We considered how these ethical issues faced on the ground mapped on to clusters of research ethics principles outlined by Emanuel et al.,^24^ given the widespread awareness of this framework in LMICs, our use of it in our previous related paper,^25^ and this framework being easy to apply for the types of HPSR we were conducting relative to others.

## Findings and discussion

The range of ethical issues we faced are illustrated in Boxes 1, 2, 3, grouped broadly into difficult information and observations (Box 1 and Box 2), and strengths and challenges of conducting research embedded in health systems (Box 3). As will be shown, many of the ethical issues we identified emerged after our research had begun, or on completion, and related to complex interactions and relationships between community members, health providers and managers, and researchers. We discuss these issues under clusters of principles summarised in Emanuel et al, and in relation to the literature. In each section we draw on the findings and discussion to make suggestions for consideration in similar future studies.

Box 1 **Illustrative examples of difficult information and observations: learning sites work in health centres and dispensaries in Kilifi County**

Researchers shadowing health facility managers for several weeks were uncomfortable when they observed apparently unethical behaviour, such as some of the poorest or most ‘illiterate’ patients being left to wait unnecessarily for services. We also found it challenging to observe patients being given apparently inadequate information or being talked to rudely. We were sometimes asked by patients to talk to the health worker or even to step in and help out with for example record filling or drug administration. Less commonly, community members openly blamed over‐stretched front‐line staff about resource shortages in facilities; accusing them of misuse of funds or drugs at a time that staff were facing significant and diverse challenges themselves. Our dilemmas included how to respond in the short term (to each event) and overall through communication of research findings to health system managers. The challenge was how to make a positive difference – ideally systematically ‐ and avoid any backlash or ‘scape‐goating’ that might exacerbate existing inequities or vulnerabilities. We were aware that even apparently small actions or responses, and indeed inaction such as failure to respond, can feed into dynamic relationships with the potential for both unexpected and unintended outcomes.

Box 2 **Illustrative example of difficult information and observations: hospital priority setting study**

We were aware from the outset of the potentially sensitive nature of efforts to understand what resources are available to hospitals, key managers’ decision‐making power over those resources, and how funds are allocated across departments. Our communication plans, including information and consent processes, were carefully and honestly worded to minimise concerns and reassure that the study is not an audit and about confidentiality. However over the course of intensive periods of fieldwork in the two hospitals, we increasingly understood the centrality of key individuals and power relations to the implementation and outcome of priority setting processes. As relationships between researchers and hospital managers developed, the lead researcher also began to hear allegations of corruption and misuse of funds, often in informal interactions with research participants. As with Box 1, dilemmas included how to appropriately respond to allegations in the short and longer term. Regarding the latter, we wanted to share important findings in such a way as to illustrate the richness and depth of data without compromising confidentiality agreements; not straightforward in contexts where many health systems managers know each other and study settings very well.

Box 3 **Examples from the learning site on strengths and challenges with embeddedness in health systems**

One of the lead researchers (BT) on this study has had continuous engagements with managers at national, county and sub‐county level in several different capacities: as head of KEMRI‐Wellcome research programme‐Ministry of Health liaison; as a formal technical advisor to the Ministry of Health at national and county level; as informal mentor and advisor at all levels given his roles; as a learning site PI and PhD student; and most recently as Director of the centre. Embeddedness for this study has had positive implications for learning about how health systems function over time, and having relationships in place that facilitate interest in and uptake of research findings into policy and practice; and therefore the social value of the work. BT for example has been consulted by senior health system managers and been able to draw on our research findings to advise on county finance policy and health sector planning. Also BT and EB hold formal national advisory level positions contributing to finance and planning policy and practice at that level. However there are also challenges with this embeddedness, including the need to be careful in who one is (seen to be) aligned with in inevitably politically charged and socially unequal contexts, which in turn influences others’ perceptions and engagements with us, our learning, and how are findings are listened to and taken up. Regarding consent processes, these are complicated by explanations of the research arguably only apply to some of the activities undertaken by BT, by the deliberate blurring between research and practice activities in some cases, and by the research team having a particular interest in accessing informal and tacit information that is not easily elicited from formal interviews.

### Informed consent and respect for recruited participants and communities

Many of the issues we faced related to our *information and consent processes* (see Box 2 and Box 3 for examples). A key challenge was that regardless of the care taken in initial explanations, there was often an assumption that we were conducting some form of audit of individuals or facilities, or – particularly for studies 2 and 3 ‐ that we would be able to fix a range of health system governance challenges.

For the HSSF study, we asked ourselves how much choice facility in‐charges really had to refuse our work: we arrived in facilities with formal letters from senior managers saying we had been given permission to come and ask them if they were willing to be in the research. We were concerned about whether they were really in a position to refuse. In reflecting on this, we wondered whether it is always appropriate to consent in‐charges for potentially important public service research in facilities, and noted Dixon‐Woods and Bosk's comment (2011):

*‘This is the distinctive dilemma that an ethics of public services research must confront: getting answers to important questions may mean insisting that public sector workers tolerate the risks and discomforts of being studied’ (p260)*.

For our more embedded studies, we worried about whether our initial formal consent processes could really cover use of information gained many months later, often in informal out of work interactions (over a meal or a drink). Relatedly for these studies, our collaborators’ understanding of our research shifted over time, and not always towards greater understanding of our research aims and ways of working. For example in the learning site, some of our collaborators moved towards seeing us more as confidantes and problem‐solvers on complex governance issues than as researchers aiming to learn about micro‐processes of governance. This was not surprising given our own evolving understanding of our role and ways of working as researchers, and in some cases – see for example Box 3 ‐ our complex positionality.

In relation to respect to participants and communities *beyond consent processes*, a key challenge was being informed about or observing apparently ‘unethical’ behaviour between health providers and community members, or among health system colleagues and managers. Possible ‘unethical’ behaviour reported or observed included for example unfair charging practices, poor or even abusive communication towards staff from patients or vice versa, other forms of sub‐optimal quality of care or management, and apparently inappropriate resource management. Boxes one and two provide illustrative examples of such dilemmas. In both cases, deciding on what is unethical, and what, if any, action is appropriate was far from simple. Firstly, reported or observed activities are often coping strategies in very difficult situations. We learned for example through our research that primary health care facility in‐charges are expected to perform complex and diverse roles in a difficult environment with relatively little formal preparation and job clarity.^26^ This while being accountable in multiple directions (to patients, colleagues, line managers and other funders) in a context of significant resource shortages, and remuneration anxieties. Secondly, decisions on what is unethical and how to respond are also complicated by each issue identified being different (in terms of potential types and amounts of harm being observed), by difficulties in knowing if and when we understand the situation well enough to act, and by lack of clarity in whether as researchers we have a right or responsibility to speak on behalf of others. This is particularly challenging given potentially negative consequences of any actions we take, including for those we are trying to assist. Such dilemmas are not unique to our studies; as Dixon‐Woods and Bosk^27^ note:

*As long as researchers make it their business to study work that has murky everyday ethical decision‐making, they have to learn to live with the ethical mess of [their own and] other people's work (p 267) insertion is our own*.

Overall we found ourselves in an ethically complex web of interactions where we were balancing several sometimes conflicting concerns: 1) avoiding introducing anxiety and discomfort among our participants (for example based on a concern that we are conducting audits aimed at identifying individual malpractice). Such discomfort risks undermining our access to key information and therefore our understanding of ground realities; 2) ensuring that we are not deceiving diverse participants about the nature and intent of our work, and what it can realistically achieve; and 3) building and maintaining respectful relationships with managers that support rather than undermine their understanding of our work, and our learning.

We discussed each issue as it arose in regular reflective practice meetings, and in informal meetings. Over time we noted a pattern: that in our responses to individual issues we were generally trying to work with individuals to support them to develop their own solutions in the hope that this would have a longer term positive impact. Also, in local level feedback meetings we were avoiding discussing individual situations and people, and providing instead broader, more generalizable lessons. Throughout, we were highlighting wherever possible positive practices to learn from and build upon.

Drawing on our experience and related literature, more specific suggestions we have for similar studies include:

- Consent in terms of fixed messages given formally to all potential participants at the outset of the study is only one part of a much wider set of interactions which show respect to diverse participants. Regular interaction, discussion and reflection with key actors wherever possible is essential.
- Individual informed consent can in some cases feel meaningless, impracticable or even create unnecessary risks.^28^ Research ethics guidance from the ESRC accepts that covert research may occasionally be appropriate, and we agree that this should not be ‘undertaken lightly or routinely’.^29^
- Ensuring that all actors in the research team understand and ‘buy into’ the research and research approach through regular formal and informal interactions is essential to honest and clear interactions, asking the right questions, building good quality relevant data, and minimising unrealistically high expectations among participants.
- There may be particular concerns regarding breach of confidentiality, where individuals, facilities or regions are easily identifiable, with negative implications for future (transformative) research. This raises the dilemma of how much information can be shared without risking participants being identified, and thereby undermining respect.

### Risk benefit ratios (ie balancing benefits and disadvantages)

There were no formal benefits built into our studies for individual participants, but we felt–and were often told–that participants enjoyed sharing their views, experiences and recommendations. Our goal was to produce new knowledge that would contribute to strengthening health systems in future, and many of our participants had expectations that sharing information with us would contribute to positive change, albeit in unclear ways. Particularly for the learning site work, potential benefits built into the study design included researchers working with health managers to identify changes in daily practice that might ultimately strengthen health systems and public health. Aiming for changes that impact positively on whole communities is important in LMICs: it is increasingly recognised that crucial ethical concerns in these settings should move beyond the micro‐level of individual rights and interpersonal relations, to include the wider interests of whole populations.^30^

We encountered many challenges in relation to risks and benefits however, including the sensitive nature of some data collected, and the potential to raise anxiety and raised expectations across all three studies. With regards to sensitive data, an example is the corruption allegations shared in Box 2, which raised dilemmas as discussed in the previous section on if and how to share such information. In relation to raised expectations, we were presented with numerous requests from health managers and providers over the course of studies. Requests ranged from researchers being asked to assist with small roles in busy facilities (Box 1) and in giving lifts in research vehicles, through requests for extra allowances, to suggestions that we support routine facility supervision activities with research vehicles, and provide funds for emergency drugs for facilities. Particularly in the learning site study, senior heath managers sometimes also requested specific information from our studies that would provide ‘independent’ or ‘scientific’ support for decisions they had already made (such as the transfer of a senior staff member). Researchers were also asked several times to step in and help ‘fix’ various management issues or challenges, such as the apparent non‐performance of a senior hospital manager or the lack of clarity and tensions in the evolving county organogram. Such requests were often raised in a highly charged context politically, because our study was being conducted in the early stages of major political devolution; a period characterised by disagreement, debate and complex power struggles.^31^

Requests introduced dilemmas for researchers. Acting on resource requests has potentially important positive implications for learning about health system realities through for example tracking how these actions work out over time, and for building relationships with managers that are crucial to HPSR. However in acting on such requests would we change what we were observing? We were also concerned about how sustainable any support we offered would be, and whether if we intervened in the short term we might undermine the possibility for future more internally developed, longer term solutions. Another dilemma was whether responses would raise expectations of what researchers can do unrealistically, and undermine understanding of our role as researchers.

In terms of information requests, we recognised that this could be an opportunity to carefully feedback research lessons at a time and in a form most needed by senior managers (and that if this research knowledge was acted upon, that this might strengthen the benefits associated with our research). However, this had to be done in a way that did not undermine key relationships in health systems, or confidentiality agreements with participants, which would have potentially negative implications for less powerful actors in health systems, as well as for the quality of our science.

An important influence on receiving and handling requests was how embedded we were as researchers in the communities and health systems we were studying. The longer and stronger the links and relationships between researchers and managers, and the more participatory and action research elements were incorporated, the more blurred the boundary between ‘researcher’ and ‘participant’, with associated strengths and challenges (Box 3). An ethical dilemma associated with studies where relationships evolve over time between researcher and participant, or where lines are blurred, is that:

*‘Researchers may come to see questionable practices as normal and acceptable, possibly because they become so acclimated to study settings, or because they feel uncomfortable about ‘betraying’ the staff who allowed them access’ (Dixon‐Woods and Bosk (2011))*.

An underlying dilemma was how to respond to requests in a way that balances: a) being protective of a diverse range of individual participants’ and facilities’ rights to confidentiality, with b) ensuring that the voices of those that are usually not included in policy and practice debates are heard by those with the potential to introduce change, and that c) any potential to transform – rather than exacerbate – unequal power relations was embraced. This is an important dilemma given that an arguably fundamentally ethical role of social science should be to challenge established orthodoxies and interests.^32^

Drawing also on the wider literature, we suggest that for studies like ours:

- Individual risks and benefits are not as obviously and automatically incorporated into HPSR in the way they often are for epidemiological or clinical studies. Careful consideration and planning is needed to ensure that relationships are not harmed, and power imbalances not exacerbated. A dilemma is how to counter‐balance this cautionary approach against a transformative agenda to raise awareness about and reduce damaging imbalances in an effort to strengthen equity. Where the intention is to improve equity between actors, there needs to be careful planning and tracking of activities over time to identify and respond to unintended perverse outcomes that undermine this intention.
- Feedback to collaborators and actors at different levels of the system, and the way in which that feedback is given, is essential to having a positive and ideally transformative impact beyond individual research participants. Often such feedback will require informal interaction, and being able to respond to requests for information and engagement positively and clearly, with very short notice.

### Social value, scientific validity and collaborative partnerships

Overall, we observed that the scientific validity (or ‘trustworthiness’ of our findings) often depended on researchers’ relationships with a range of different health system actors, all of whom are part of complex health systems imbued with unequal and shifting power‐relations. For example many users appeared relatively powerless vis a vis providers, and many providers less powerful than their line managers and political actors. Nevertheless, at all levels we also observed providers and managers ‘going the extra’ mile to provide care in challenging environments and agency being overtly or covertly exercised by those with less obvious power, with both positive and negative implications for health delivery and equity.

Research staff have the potential to become part of these complex relationships and to influence them in both intended and unintended/unexpected ways. For the longer term more embedded studies we aimed to work with some of these actors as partners rather than interviewees. Building appropriate relationships with these partners and other health system actors is essential not only to making sure the right research questions are asked, but also to how much of these individuals’ knowledge is accessible to researchers, particularly their tacit knowledge. Relationship building takes time and can be unpredictable, especially where key actors are constantly shifting positions, as is often the case in health systems. There is also a fundamental dilemma in building these relationships and understandings, as noted above, that we remain able to question norms, values and practices, and contribute ultimately to the transformation of inappropriate practices and fundamental inequities.

In our case, we again found that regular honest reflective practice sessions among the very diverse researcher team was invaluable. In deliberating on each researchers’ position, dilemmas faced, how these were handled and implications for our learning, we observed how critical positionality and reflection on that is; that differences between the researcher(s) and key actors in race, class, nationality, gender or education, together with the attitudes and communication skills of researchers, can influence openness, honesty, and our ability to step back and see the wood from the trees. This has important implications for both science and ethics, which are inevitably intertwined.

### Independent ethical review

As noted above, all three studies were reviewed and approved in advance with no major scientific or ethical issues raised. We were careful in writing proposals to clarify that consent forms and processes would be adapted for each participant, and that questions and tools would evolve over time.

The ethical issues that did emerge over time – largely linked to diverse and complex social relationships – were given little emphasis in the initial study protocols. Although these relational aspects of our research were critical to ethics practice, they are not easily tested and checked by ethics committees in advance, particularly where committees most familiar with reviewing biomedical studies. In fact we are uncertain how these essential elements ‐ linked as they are to the insight and integrity of all involved in the research endeavour ‐ can be checked by national committees at all, at least when ethics committees function in a procedural way. Relational elements can also unfold over the course of research in ways that can be difficult or impossible to predict in advance, and that require recognition and appropriate response as they arise.

For social science research conducted in LMICs there is a debate as to whether ethics review processes are critical to ensuring national interests are considered, global inequities challenged and research questions and processes tailored to local contexts, or whether these often highly procedural processes essentially narrow the scope of ethical reflection and depoliticise ethical debates through encouraging ticking off of ‘correct procedures’.^33^ There is a concern that these processes also undermine critical, transformative social science through requiring tools. As noted by Dixon‐Woods and Bosk (2011) we need to be careful that such institutionalized forms of research ethics, designed to protect the right of vulnerable subjects, are not used to strategically defend the privileges of the powerful. They argue for a nuanced and context specific form of independent oversight that both defends against poor social science, and that recognises and values researchers’ critical skills and moral commitments.

In our context, deliberative spaces for reflection among the research team were invaluable, as noted above; supported by external collaborators conducting similar work in other settings. For the learning site, given the more evolving and embedded study design, we incorporated more independent voices into these reflections through submitting detailed annual reports to external experts, in addition to filling routine annual reports required by the national ethics committee. In our internal reflective practice sessions, we could consider ourselves as building skills in being more ethically minded in our daily activities. Guillemin and Heggen^34^ argue that researchers can build ethical mindfulness by: (1) acknowledging the role of ethically important moments in the everyday practice of research; (2) giving credence to ‘not feeling quite right’ about a research situation; (3) articulating what is ethically important in the practice of research through application of the principles of respect, justice and beneficence; (4) being reflexive, that is, taking stock of actions and their role in research; and (5) having courage by way of being receptive to new ways of thinking about research ethics and critically challenging established research practice. We feel we were beginning to touch on all of these elements in our reflexive practice. However we would like to draw on this and related work to learn more about how we can build up our ethical mindfulness for future similar studies, and incorporate well‐informed but independent inputs into our reflections.

## Conclusion

In this paper, we examined the ethical issues that arose over the course of three primarily qualitative governance studies in Kenya in which health managers and providers at the district, county or facility level were key participants. We show that many of the ethical dilemmas and challenges we faced only emerged over the course of the fieldwork. Many were related to the social relationships – often involving complex imbalances of power ‐ that are established in research teams, between researchers and health staff and managers, and between field‐teams and community members. These relational elements of our research are often critical to ethics practice, and to conducting quality science, but are not easily tested and checked by ethics committees more familiar with reviewing biomedical studies. Many of the issues raised also point to the range of challenges that frontline health managers and workers face in LMICs, and to the importance of stewardship of health systems and the ethical responsibility that governments have in facilitating the delivery of services to populations.

We support others in the continued drive towards identifying and conceptualising key ethical issues and appropriate oversight of health systems research,^35^ and in arguments that we should be working towards a more nuanced and context specific form of independent oversight that both defends against poor HPSR, and that recognises and promotes researchers’ critical skills and moral commitments.^36^ We also recognise that in our day‐to‐day practice, we have a responsibility to build up our ‘ethical mindfulness’.^37^ Many of these essential relational elements of ethical practice, and of producing quality data, are given stronger emphasis in social science research ethics than in biomedical ethics,^38^ and are particularly relevant for HPSR where health systems are understood as social and political constructs with vital opportunities for tackling social injustice.^39^ They are however likely to be highly relevant to conducting many types of research all over the world.

## Notes

1 Evans T, Nishtar S, Atun R, et al. Scaling up research and learning for health systems: time to act. Lancet 2008;372(9649):1529‐31.

2 Kieny MP, Evans DB, Schmets G, et al. Health‐system resilience: reflections on the Ebola crisis in western Africa. Bulletin of the World Health Organization 2014;92(12):850

3 Remme JH, Adam T, Becerra‐Posada F, et al. Defining research to improve health systems. PLoS medicine 2010;7(11):e1001000.

4 Sheikh K, Gilson L, Agyepong IA, et al. Building the field of health policy and systems research: framing the questions. PLoS medicine 2011;8(8):e1001073.

5 Sheikh K, George A, Gilson L. People‐centred science: strengthening the practice of health policy and systems research. Health research policy and systems / BioMed Central 2014;12:19.

6 Ibid.

7 Hyder AA, Pratt B, Ali J, et al. The ethics of health systems research in low‐ and middle‐income countries: a call to action. Glob Public Health 2014;9(9):1008‐22.

8 Shore N, Wong KA, Seifer SD, et al. Introduction to special issue: advancing the ethics of community‐based participatory research. Journal of empirical research on human research ethics: JERHRE 2008;3(2):1‐4.

9 Macklin R. Ethical Challenges in Implementation Research. Public Health Ethics 2014;1(8).

10 Weijer C. The Ottawa Statement on the Ethical Design and Conduct of Cluster Randomized Trials. PLoS medicine 2012;9(11):e1001346. doi:10.1371/journal.pmed.46.

11 Faden R. An Ethics Framework for a Learning Health Care System: A Departure from Traditional Research Ethics and Clinical Ethics. . Hastings Centre Report 2013;43((s1)):S16‐S27.

12 Hyder AA, Rattani A, Krubiner C, et al. Ethical review of health systems research in low‐ and middle‐income countries: a conceptual exploration. The American journal of bioethics : AJOB 2014;14(2):28‐37.

13 Ibid.

14 Hyder AA, Pratt B, Ali J, et al. The ethics of health systems research in low‐ and middle‐income countries: a call to action. Glob Public Health 2014;9(9):1008‐22; Hyder AA, Rattani A, Krubiner C, et al. Ethical review of health systems research in low‐ and middle‐income countries: a conceptual exploration. The American journal of bioethics : AJOB 2014;14(2):28‐37.

15 Molyneux S, Geissler PW. Ethics and the ethnography of medical research in Africa. Social science & medicine (1982) 2008;67(5):685‐95.

16 Molyneux CS, Goudge J, Russell S, Chuma J, Gumede T and L Gilson (2009). Conducting health‐related social science research in low income settings: ethical dilemmas faced in Kenya and South Africa. Journal of International Development, 21, 309–326.

17 Emanuel EJ, Wendler D, Killen J, et al. What makes clinical research in developing countries ethical? The benchmarks of ethical research. The Journal of infectious diseases 2004;189(5):930‐7.

18 Wassenaar DR, Mamotte N. Ethical issues and ethics reviews in social science research. In: Leach M, Stevens M, Lindsay G, et al., eds. The Oxford handbook of international psychological ethics. New York: Oxford, 2012:268‐ 82.

19 Hoeyer K, Dahlager L, Lynoe N. Conflicting notions of research ethics. The mutually challenging traditions of social scientists and medical researchers. Social science & medicine (1982) 2005;61(8):1741‐9.

20 Scott V, Schaay N, Olckers P, et al. Exploring the nature of governance at the level of implementation for health system strengthening: the DIALHS experience. Health policy and planning 2014;29(suppl 2):ii59‐ii70. Topp SM, Chipukuma JM, Hanefeld J. Understanding the dynamic interactions driving Zambian health centre performance: a case‐based health systems analysis. Health policy and planning 2014.

21 Waweru E, Goodman C, Kedenge S, Tsofa B, Molyneux S. Tracking implementation and (un)intended consequences: a process evaluation of an innovative peripheral health facility financing mechanism in Kenya. Health policy and planning. 2016 Mar 1; 31(2):137‐47.

22 Elloker S, Olckers P, Gilson L, et al. Crises, routines and innovations: the complexities and possibilities of sub‐district management: leadership and governance. South African Health Review 2012:161‐73. Nyikuri M, Tsofa B, Barasa E, Okoth P, Molyneux S. Crises and Resilience at the Frontline—Public Health Facility Managers under Devolution in a Sub‐County on the Kenyan Coast. PloS one. 2015 Dec 22;10(12):e0144768.

23 Authors included Sassy Molyneux, Benjamin Tsofa, Edwine Barasa, Mary Nyikuri, Evelyn Wanjiku Waweru, and Catherine Goodman.

24
*Op. cit*. note 17.

25
*Op. cit*. note 16.

26 Nyikuri et al., *Op. cit*. note 22.

27 Dixon‐Woods M, Bosk CL. Defending Rights or Defending Privileges? Public Management Review 2011;13(2):257‐72.

28 Ibid.

29
*Op. cit*. note 28. Council. EaSR. Developing a framework for social science research ethics. 2015.

30 Benatar SR. Reflections and recommendations on research ethics in developing countries. Social science & medicine (1982) 2002;54(7):1131‐41. Bhutta ZA. Ethics in international health research: a perspective from the developing world. Bulletin of the World Health Organization 2002;80(2):114‐20. Molyneux S, Mulupi S, Mbaabu L, et al. Benefits and payments for research participants: Experiences and views from a research centre on the Kenyan coast. BMC medical ethics 2012;13:13. Pratt B. Connecting health systems research ethics to a broader health equity agenda. The American journal of bioethics : AJOB 2014;14(2):1‐3.

31 Nyikuri et al., *Op. cit*. note 22.

32
*Op. cit*. note 26.

33
*Op. cit*. note 15.

34 Guillemin M, Gillam L. Ethics, Reflexivity, and “Ethically Important Moments” in Research. Qualitative Inquiry 2004;10(2):261‐80.

35 Hyder AA, Pratt B, Ali J, et al. The ethics of health systems research in low‐ and middle‐income countries: a call to action. Glob Public Health 2014;9(9):1008‐22. Hyder AA, Rattani A, Krubiner C, et al. Ethical review of health systems research in low‐ and middle‐income countries: a conceptual exploration. The American journal of bioethics : AJOB 2014;14(2):28‐37.

36 Dixon‐Woods M, Bosk CL. Defending Rights or Defending Privileges? . Public Management Review 2011;13(2):257‐72. Dixon‐Woods M, Bosk C. Learning through observation: the role of ethnography in improving critical care. Current opinion in critical care 2010;16(6):639‐42.

37
*Op. cit*. note 34.

38 Rogers J, Kelly UA. Feminist intersectionality: bringing social justice to health disparities research. Nurs Ethics 2011;18(3):397‐407. Tong R. Feminist approaches to bioethics. J Clin Ethics 1996;7(4):315‐9. DeRenzo EG. Power differentials, context, relationship, and emotions: feminist ethics considerations and human subjects research. J Womens Health 1998;7(8):971‐7.

39 Sheikh K, George A, Gilson L. People‐centred science: strengthening the practice of health policy and systems research. Health research policy and systems / BioMed Central 2014;12:19.

